# Complete mitochondrial genome sequence of grey-headed parrotbill (*Paradoxornis gularis*) and its phylogenetic analysis

**DOI:** 10.1080/23802359.2019.1674203

**Published:** 2019-10-21

**Authors:** Weiqiang He, Huailiang Xu, Diyan Li, Meng Xie, Mingwang Zhang, Qingyong Ni, Yongfang Yao

**Affiliations:** a College of Life Science, Sichuan Agricultural University, Ya’an, Sichuan, China;; b College of Animal Science and Technology, Sichuan Agricultural University, Chengdu, Sichuan, China

**Keywords:** Mitochondrial genome, *Paradoxornis gularis*, phylogenetic analysis

## Abstract

Grey-headed Parrotbill, *Paradoxornis gularis*, has significant economic and scientific value in China. Length of the complete mitochondrial genome of grey-headed parrotbill is 17,743 bp, and it contains 13 protein-coding genes (PCGs), 2 ribosomal RNA genes (*12S rRNA* and *16S rRNA*), 22 transfer RNA genes (tRNA), and a D-loop sequence. The overall base composition of the mitochondrial sequence was 29.47% A, 14.68% G, 24.33% T, 31.52% C. All tRNAs have the typical cloverleaf structure, except for *tRNA^Ser^*. All PCGs start with ATG codons, except for *COXI* and *NADH6*, which are initiated by GTG and CTA, respectively.

Grey-headed parrotbill (*Paradoxornis gularis*), with significant economic and scientific value in China, is one of bird species often placed with the Old World babblers (family Timaliidae) or in a distinct family Paradoxornithidae, but it actually seems to belong to the Sylviidae. The body’s length is about 18 cm, grey overhead with black stripes, an orange beak, a red iris, grey feet, and it is distributed in China, Bhutan, India, Lao People’s Democratic Republic, Myanmar, Thailand, and Vietnam. This species has an extremely large range, but the global population size has not been quantified. Therefore, this species is evaluated as Least Concern (BirdLife International 2016). In this study, we sequenced and analyzed the complete mitochondrial of grey-headed parrotbill to provide more useful information for bird research.

The sample was collected from Ya’an (30°0′47.20″N, 103°02′25.51″E) Sichuan province of China and stored at the Zoology Laboratory, Sichuan Agricultural University (Accession no.: 000878), China. Total DNA of grey-headed parrotbill was extracted from muscle tissue by the traditional phenol-chloroform methods (Sambrook 2001). Then, 15 samples were designed and used to amplify the overlapping segment of its mitochondria and splicing sequences. Software DNAstar was used to analyze the complete mitochondrial genome of grey-headed parrotbill (Burland 2000). The mitochondrial genome sequences of grey-headed parrotbill have been deposited in NCBI under accession number MK900637.

The length of grey-headed parrotbill sequence is 17,743 bp and contains 13 protein-coding genes (PCGs), 22 transfer RNA genes (tRNA), 2 ribosomal RNA genes (*12S rRNA* and *16S rRNA*) and a D-loop region, which was consistent with the other studies of Passeriformes (Yang 2016). The overall base composition of the mitochondrial sequence was 29.47% A, 14.68% G, 24.33% T, 31.52% C and the length of tRNA range from 68 to 76 bp. *NADH6* and eight tRNA genes were transcribed from the L-strand, the remaining genes were encoded on the H-strand. All the tRNAs have the typical cloverleaf structure, except for *tRNA^Ser^*, which lacks the arm of dihydrouracil (DHU). All PCGs start with ATG codons, except for *COXI* and *NADH6*, which are initiated by GTG and CTA, respectively. There are two types of stop codons; complete and incomplete. Interestingly, *NADH1* and *COXI* shared stop codon *AGG*, *NADH6* stopped with CTA. Similar to previous research results, *12S rRNA* and *16S rRNA* genes were located between the *tRNA^Phe^* and *tRNA^Lue^*, separated by *tRNA^Val^* (Queiroz 2017; Hsieh et al. 2018).

MEGA10.0 (Kumar et al. 2018) was used to construct a phylogenetic tree based on whole complete mitochondrial sequences including 11 species and *Aptenodytes forsteri* was used as one outgroup (Figure 1). All species were clustered into three clades; Muscicapidae, Sylviidae, Timaliidae, and *P. gularis* was the sister clade of *Paradoxornis fulvifrons.*

**Figure 1.**
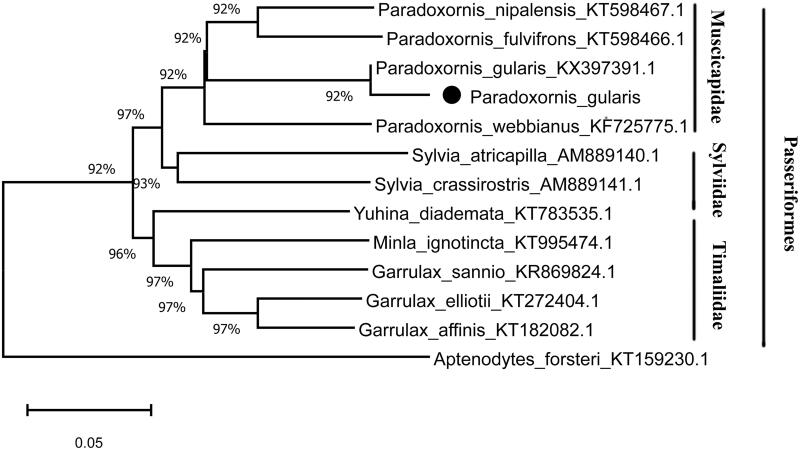
Phylogenetic tree of *Paradoxornis gularis* based on the neighbor-joining analysis and *Aptenodytes forsteri* was used as one outgroup. Black dots represent a sequence of this study.

## References

[CIT0001] BirdLife International 2016 *Psittiparus gularis* The IUCN Red List of Threatened Species. [Cambridge (UK)]: BirdLife International; [accessed 2019 June 18]. 10.2305/IUCN.UK.2016-3.RLTS.T22735202A95105743.en.

[CIT0002] Burland TG 2000 DNASTAR’s Lasergene sequence analysis software. Methods Mol Biol. 132:71–91. 10547832 10.1385/1-59259-192-2:71

[CIT0003] Hsieh H-I, Hou H-Y, Chang R-X, Cheng Y-N, Jang-Liaw N-H 2018 Complete mitochondrial genome sequence for the Taiwan Blue Magpie *Urocissa caerulea* (Passeriformes: Corvidae). Mitochondrial DNA Part B. 3:665–667. 10.1080/23802359.2018.1481778 PMC7799675 33474277

[CIT0004] Kumar S, Stecher G, Li M, Knyaz C, Tamura K 2018 MEGA X: Molecular Evolutionary Genetics Analysis across computing platforms. Mol Biol Evol. 35:1547–1549. 29722887 10.1093/molbev/msy096 PMC5967553

[CIT0005] Queiroz S 2017 Complete mitochondrial genome of *Sporophila maximiliani* (Ave, Passeriformes). Mitochondrial DNA Part B. 2:417–418. 10.1080/23802359.2017.1347840 PMC7800134 33473846

[CIT0006] Sambrook JR 2001 Molecular cloning: a laboratory manual. 3rd ed New York (NY): Cold Spring Harbor Laboratory Press; p. 12–72.

[CIT0007] Yang G 2016 The complete mitochondrial genome sequence of Eophona migratoria (Passeriformes Fringillidae). Mitochondrial DNA Part B. 1:753–754. 10.1080/23802359.2016.1209098 PMC7799522 33473615

